# Sexual Dysfunctions and Their Association with the Dual Control Model of Sexual Response in Men and Women with High-Functioning Autism

**DOI:** 10.3390/jcm8040425

**Published:** 2019-03-28

**Authors:** Daniel Turner, Peer Briken, Daniel Schöttle

**Affiliations:** 1 Department of Psychiatry and Psychotherapy, University Medical Center Mainz, 55131 Mainz, Germany; 2 Institute for Sex Research and Forensic Psychiatry, University Medical Center Hamburg-Eppendorf, 20246 Hamburg, Germany; briken@uke.de (P.B.); d.schoettle@uke.de (D.S.); 3 Department of Psychiatry and Psychotherapy, University Medical Center Hamburg-Eppendorf, 20246 Hamburg, Germany

**Keywords:** sexual dysfunction, autism, erectile dysfunction, sexual satisfaction, Asperger syndrome, sexual desire, lubrication, sexual intercourse, sexual excitation, sexual inhibition

## Abstract

Adults with an Autism Spectrum Disorder (ASD) are characterized by impairments in social interaction and communication, repetitive and stereotyped interests and behaviours as well as hyper- and/or hyposensitivities. These disorder specific symptoms could be associated with the development of sexual disorders. The Dual Control Model of Sexual Response presents one approach that is frequently used to explain the emergence of sexual dysfunctions. The aim of the present study was to assess the extent of symptoms of sexual dysfunctions in men and women with ASD and to evaluate their association with the individual propensity of sexual excitation and inhibition as defined by the Dual Control Model. Both men and women with ASD were more likely to report about sexual dysfunctions than individuals from the control group. In men with ASD, sexual inhibition was significantly correlated with the emergence of sexual dysfunctions, while there was no association between sexual functioning and sexual excitation. In women, the opposite pattern was found. Especially the peculiarities in sensitive perception could be responsible for the observed problems with sexual functioning in individuals with ASD. The present findings highlight the great need for specialized treatment programs addressing the frequently observed sexuality-related problems in individuals with ASD. However, up to now such treatment programs are lacking.

## 1. Introduction

Autism Spectrum Disorder (ASD) is characterized by impairments in social interaction and communication, as well as repetitive and stereotyped interests and behaviours [1]. It is estimated that up to 1.7% of the population are affected by ASD [2,3]. About 50% of individuals with ASD have average intellectual functioning and in the meantime more and more adults are being diagnosed in later life [4]. Just like in other neurodevelopmental disorders, there is a male preponderance in ASD and the male to female ratio is estimated to be around 3–4:1 [5,6]. However, these reported gender differences are currently subject of a controversial discussion and it is suggested that this effect might be largely attributable to the possible gender-biased artefact of a male-symptomatic based diagnostic system with later diagnosed females requiring heavier symptom loads for diagnosis [7].

Nevertheless, all individuals with ASD have in common that they have (in varying degrees) difficulties in interpreting non-verbal cues, such as decoding and interpreting facial expressions and have limited capabilities in theory of mind skills [1]. When throughout development social interactions become more complex and romantic and sexual relationships become increasingly important, the learned social skills often cannot keep up with the social demands needed for the initiation and maintenance of romantic peer-relationships [8]. Thus, many stereotypes around individuals with ASD concerning sexuality related issues have arisen, such as, ASD individuals are seen as being only sparsely interested in sexual and romantic relationships or as being mainly asexual [9,10]. Contrary to these stereotypes, however, in recent years a growing body of research has accumulated showing that most individuals with ASD report a general interest in solitary and dyadic sexual behaviours and show the full range of sexual behaviours, just like their clinically non-affected counterparts [11,12,13,14]. Nevertheless, the deficits in intuitively understand social and nonverbal communication cues, difficulties in perspective-taking, inflexibility, affective dysregulation, repetitive and stereotyped interests and peculiarities in sensitive perception leading to either over- or underreactions to sensory stimuli can hamper the development of romantic and sexual relationships, can be associated with impaired sexual functioning and sometimes also with the development of sexual disorders [15,16,17].

In a first study of our working group, focusing on paraphilias and hypersexuality in high-functioning men and women with ASD, it was found that high-functioning ASD men reported more frequently about masochistic, sexually sadistic, voyeuristic, frotteuristic and paedophilic fantasies and more frequently about frotteuristic behaviours compared to men from a control group. Furthermore, more men with ASD reported about hypersexual fantasies and behaviours than their non-affected peers. High-functioning ASD women reported more frequently about masochistic behaviours than healthy women, while no other differences occurred [18]. Men with ASD usually show a more pronounced ASD symptomatology regarding for example, repetitive behaviours or hypo- and hypersensitivities, which could be one possible explanation for the higher prevalence of hypersexual and paraphilic fantasies and behaviours in ASD men compared to ASD women [18,19]. Thereby, the more frequently observed repetitive behaviours and obsessive interests could translate into sexualized interests and behaviours, which result in a faster habituation leading the individual to seek novel sexual activities, for example, paraphilic sexual activities.

Besides paraphilic and hypersexual behaviours, the disorder-inherent deficits and symptoms could also be accompanied by sexual dysfunctions in ASD men and women. Sexual dysfunctions are disorders characterized by a clinically significant disturbance in a person’s ability to respond sexually or to experience sexual pleasure [1]. Sexual dysfunctions are usually classified in accordance with the four phases of the sexual reaction cycle: disorders of sexual appetence (e.g., female sexual interest/arousal disorder), disorders of sexual desire (e.g., erectile disorder, male hypoactive sexual desire disorder), orgasm disorders (e.g., premature (early) ejaculation and delayed ejaculation, female orgasmic disorder) and sexual pain disorders. (e.g., genito-pelvic pain/penetration disorder). In the general population it is estimated that about 40% to 50% of all women report at least one sexual dysfunction throughout their lifetime, while the life-time prevalence in men is estimated to be about 20% to 40% [20,21,22].

Bancroft and Janssen have developed a theoretical model, which could help to explain some aspects of the emergence of sexual dysfunctions in both men and women: the Dual Control Model of Sexual Response [23]. The Dual Control Model postulates that whether or not a sexual response occurs in a particular situation depends on the interaction between an excitatory and an inhibitory neuroanatomical and neuroendocrinological network [23,24]. Individuals high in sexual inhibition and low in sexual excitation are more likely to develop sexual dysfunctions [23,25,26].

Based on these findings we aimed at assessing symptoms of sexual dysfunctions in men and women with ASD using standardized assessment scales and at evaluating the association between the individual propensity of sexual excitation and inhibition and sexual dysfunctions in both ASD individuals and healthy controls. Due to the above-stated ASD specific symptoms we hypothesized that (1.) both men and women with ASD would show more signs of sexual dysfunctions than healthy controls and (2.) that in individuals with ASD as well as in healthy controls higher sexual excitation and lower sexual inhibition scores would be related with less signs of sexual dysfunctions.

## 2. Materials and Methods

### 2.1. Participants

The present study included *n* = 96 adults with high-functioning Autism or Asperger syndrome who were compared to *n* = 96 healthy controls. In order to control for the influence of age and education on the sexual outcome measures the participants were matched concerning these variables (Table 1). All patients with ASD self-reported that they had been diagnosed by an experienced psychiatrist or psychologist. However, due to data protection regularities we did not gather any more information from the diagnosing clinicians about the diagnostic procedures. In Germany, mental disorders are usually diagnosed based on the diagnostic criteria of the International Classification of Diseases, 10th version (ICD-10) of the World Health Organization (WHO) and thus it could be assumed that all diagnoses of our study participants were made according to the ICD-10. Mean age at which patients received their ASD diagnosis was 35.7 years (SD = 9.1 years; range = 17 to 55 years). To assess the extent of autism symptoms all participants rated the German version of the Autism Spectrum Quotient-Short Form (AQ-SF) [27]. ASD patients had significantly higher scores on the AQ-SF than the healthy controls (Table 1). While all of the ASD patients scored above the proposed cut-off value of 17 points in the AQ-SF, none of the healthy controls did so. Both, the ASD individuals as well as our control participants had on average 12 years of school education suggesting that all of them had at least average intellectual functioning.

Of the ASD patients 78.2% (*n* = 75) indicated being exclusively or predominantly heterosexual, 10.4% (*n* = 10) being exclusively or predominantly homosexual, 8.3% (*n* = 8) being equally hetero- and homosexual and 3.1% (*n* = 3) indicated having no sexual orientation. In contrast, all healthy controls (HCs) were exclusively or predominantly heterosexual. Sexual orientation was assessed using the Kinsey scale [28]. More HCs (*n* = 78, 81.3%) were currently in a relationship than individuals with ASD (*n* = 27, 28.1%; *p* < 0.001) and more HCs (*n* = 96, 100%) indicated that they had previously been in a relationship lasting more than three month than individuals with ASD (*n* = 60; 62.5%; *p* < 0.001).

The control participants consumed alcoholic beverages on a more regular basis than the ASD individuals. On the other side more individuals with an ASD reported about psychiatric comorbidities, about regular medication intake in general and intake of psychopharmacological drugs in specific.

### 2.2. Procedure

All information about study participants were gathered using self-report questionnaires. These could be answered at home. Individuals diagnosed with ASD were recruited via self-help groups throughout Germany and through the Autism outpatient centre at the University Medical Center Hamburg-Eppendorf, Germany. Healthy controls were recruited through advertisements at the University Medical Center Hamburg-Eppendorf, at the University Medical Center Mainz, at local shopping malls and through personal contacts of the principal investigators.

The ethical review board of the Hamburg Medical Council approved the study protocol of the present study (PV4380).

### 2.3. Measures

#### 2.3.1. International Index of Erectile Function (IIEF)

The IIEF consists of 15 items and assesses the extent of sexual problems in male respondents. Thereby, sexual functioning is measured on five subscales: erectile functioning, orgasmic functioning, sexual desire, satisfaction with sexual intercourse and overall sexual satisfaction. Lower scores on each subscale represent more problems. The guidelines on the clinical application of the IIEF recommend that patients with a score below 14 out of 30 points on the erectile functioning subscale should be considered for treatment with Sildenafil [29]. Internal consistency for the total score as well as for all subscales of the original version of the IIEF was between α = 0.73 and 0.91 [29]. In the validation study of the German version of the questionnaire internal validity of the total score was α = 0.95, however, in contrast to the English version only a four-factorial solution was found [30]. In a follow-up study with 261 German men the original five factor structure could be replicated by confirmatory factor analysis, although a four-factor model represented an acceptable fit as well [31]. Nevertheless, in the present study we followed the original five-factor model of the questionnaire.

#### 2.3.2. Female Sexual Function Index (FSFI)

The FSFI consists of 19 items and assesses the extent of sexual problems in women on six domains: sexual desire, sexual arousal, lubrication, orgasm, sexual satisfaction and sexual pain. Lower scores represent more problems. Internal consistency for the total score as well as for all subscales of the original version of the IIEF was above α = 0.82 and test-retest reliabilities were between r = 0.79 and 0.86 for the subscales [32]. The German validation study was performed using an online sample of 1243 German women and supported the six factorial design of the original version. Internal consistencies were between α = 0.75 and 0.95 for the total score and the scores of the subscales [33].

#### 2.3.3. Sexual Inhibition/Sexual Excitation Scales-Short Form (SIS/SES-SF)

Based on the Dual Control Model of Sexual Response the SIS/SES-SF is a 14-item questionnaire that assesses participants’ reactions in sexual situations on three subscales: one sexual excitation subscale (SES) and two sexual inhibition subscales (SIS1 and SIS2) [34]. While SIS1 measures sexual inhibition due to a threat of performance failure, SIS2 assesses sexual inhibition due to a threat of performance consequences, for example, unwanted pregnancy or sexually transmitted diseases [35]. In a first validation study of the German version of the SIS/SES-SF, internal consistencies of *α* = 0.82 for SES, *α* = 0.60 for SIS1 and *α* = 0.70 for SIS 2 were reported [36].

## 3. Results

### 3.1. Relationship and Sexual Satisfaction

While more women from the control group viewed sexuality as an important part in their lives (HCs: 53.8% vs. ASD: 20%), no differences occurred concerning relationship and sexual satisfaction between women with ASD and women from the control group. Moreover, more female controls rated themselves as being sexually attractive (HCs: 53.8% vs. ASD: 20%).

When comparing ASD men to men from the control group, it was found that more male controls were satisfied with their current relationship (HCs: 63.8% vs. ASD: 11.1%) and sexual life (HCs: 59.6% vs. ASD: 10.7%). Furthermore, more male controls viewed themselves as being sexually attractive (HCs: 73.7% vs. ASD: 3.6%), while no differences occurred concerning the importance of sexuality.

Finally, when comparing ASD women with ASD men it was found that more ASD women were currently in a relationship (women: 46.2% vs. men: 16.1%), more ASD women were satisfied with their current relationship (women: 44.4% vs. men: 11.1%) and ASD women viewed themselves as more sexually attractive than ASD men (women: 20.0% vs. men: 3.6%). On the other side more ASD men viewed sexuality as an important part in their life (women: 20.0% vs. men: 50.0%). No differences occurred concerning sexual satisfaction.

### 3.2. Sexual Dysfunctions

The female controls scored significantly higher on all FSFI subscales indicating that they reported less problems with sexual desire, sexual arousal and sexual satisfaction, lubrication, orgasm quality and less sexual pain compared to the women with ASD (Table 2). The male controls also reported about significantly better overall sexual functioning than the ASD men. However, when assessing the IIEF subscales this accounted only for erectile functioning and sexual intercourse satisfaction, while no differences were found concerning orgasmic functioning and sexual desire. Furthermore, more ASD men than male controls were below the cut off for erectile functioning problems justifying the use of medication to treat these problems, however, this difference only closely approached the intended level of significance (Table 2).

In order to address the impact of the assessed clinical characteristics on sexual functioning in our ASD participants, we calculated two linear logistic regression analyses (one for the male ASD participants and one for the female ASD participants) with overall sexual functioning (IIEF sum score in men and FSFI sum score in women) as the outcome variable and regular alcohol or illegal drug intake, any psychiatric disorders, any endocrine disorders, genital abnormalities, regular intake of psychopharmacological agents and hormone replacement therapy as predictors. Table 3 gives an overview about the results of the logistic regression analyses showing that neither in ASD men nor in ASD women any of the additionally assessed clinical features had a significant influence on the overall sexual functioning scores.

### 3.3. Sexual Excitation and Sexual Inhibition

Table 2 also provides an overview about the SIS/SES-SF scores in ASD women and men compared to the HCs. While ASD women had significantly lower scores in sexual excitation compared to their non-ASD counterparts, ASD men had significantly higher scores on the sexual excitation subscale. Furthermore, women with ASD also had higher scores in SIS1 and SIS2, while no differences occurred between ASD men and the HCs.

### 3.4. Correlational Analyses

Women with ASD scoring higher on SES reported fewer overall problems with sexual functioning (Table 4). More specifically, higher SES scores were correlated with fewer problems with sexual desire, sexual arousal, lubrication and orgasm. No significant correlations were found between SIS1 and SIS2 and any of the FSFI subscales in ASD women. Comparably, in healthy women SES was also positively correlated with overall sexual functioning. Furthermore, SIS2 was negatively correlated with sexual desire and sexual arousal, meaning that those with higher SIS2 scores reported about more problems with sexual desire and sexual arousal.

In the male controls higher scores in SES were correlated with higher scores with overall sexual functioning, erectile functioning, sexual desire and overall sexual satisfaction (Table 5). In contrast, no association was found between SES and any of the IIEF subscales in ASD men, however, SIS1 and SIS2 were negatively correlated with overall sexual functioning as well as most of the IIEF subscales.

## 4. Discussion

To our knowledge, this is the first study to explore symptoms of sexual dysfunctions using self-report scales in a cohort of women and men with high-functioning ASD in comparison with a matched control group. In line with previous research, significantly less ASD men and women were currently in a romantic relationship compared to the HCs [37,38]. As was suggested in the introduction the disorder-specific symptoms like deficits in intuitively understanding social and nonverbal communication cues, difficulties in perspective-taking, cognitive and behavioural inflexibility as well as affective dysregulation, might hamper the initiation of romantic relationships in ASD individuals. Furthermore, both men and women with ASD reported lower relationship and sexual satisfaction than the HCs [39]. Within the present study we did not evaluate whether or not the current spouse of our study participants was diagnosed with ASD as well, however, this seems to be quite important, because it was shown that having a relationship with another autistic individual leads to an improved relationship satisfaction [13]. Women with ASD often have better social learning abilities, share more common interests with their peer group, have more advanced coping strategies and show less overt restricted interests and repetitive behaviours [40,41]. Thus, their problems in initiating and maintaining a romantic relationship are often not as pronounced as in ASD men, explaining why more women with ASD than men within the present study were in a romantic relationship [42]. Although fewer men with ASD were in a relationship compared to female ASD individuals, more men reported that sexuality was an important part in their life. These unfulfilled sexual desires could indicate that overall ASD men experience more distress concerning their own sexuality than ASD women.

Concerning sexual functioning it was found that men with ASD reported more problems with erectile functioning than the HCs. However, despite the findings of previous research that men with erectile dysfunctions from the general population usually have lower SES scores than those without erectile dysfunctions, the ASD men had significantly higher scores in sexual excitation than their non-affected counterparts [23,25,26]. This quite unexpected result could be the consequence of the peculiarities in sensitive perception in ASD men. On the one side the hypersensitivities experienced by many ASD men could cause that discrete (and even non-sexual) cues could be perceived as quite intense and sexually arousing, meaning that ASD men get sexually aroused more easily. In terms of the Dual Control Model this could be translated to a higher sexual excitation (e.g., Item 1 of the SIS/SES-SF: “When a sexually attractive stranger accidentally touches me, I easily become aroused”). On the other side, as quickly as sexual arousal might arise in ASD men, it could also decline again because due to the pronounced hypersensitivity a constant and increasingly strong stimulation is necessary in order to hold sexual arousal on an adequate level. This in many cases might not be possible and thus in the long run men with ASD experience more problems with erectile functioning because of the possibly more rapidly decreasing sexual arousal during (sexual) stimulation. Supporting this line of argument, the individual propensity of sexual excitation did not correlate neither with the IIEF sum score nor with any of the IIEF subscales, suggesting that sexual excitation might refer to a different kind of behaviour in ASD men compared to healthy men. A further possible explanation could be that ASD men have difficulties in recognizing and classifying signs of excitement and therefore answered the questions regarding excitement in a different manner than the male controls. Concerning the individual propensity of sexual inhibition, no differences were found between ASD men and the male controls. Furthermore, medium to large correlations in the expected direction were found between both sexual inhibition factors and the IIEF sum score and most of the IIEF subscales in the male ASD sample. These findings indicate that just like their non-affected counterparts, ASD men with a stronger propensity of sexual inhibition due to a threat of performance failure or due to a threat of performance consequences report about more sexual dysfunctions [23,26].

Comparably to the ASD men, the ASD women also reported significantly more sexual dysfunctions across all of the FSFI domains compared to the female controls. Just like in the ASD men this could be the consequence of the peculiarities in sensitive perception. However, the significantly lower sexual excitation and significantly higher sexual inhibition scores suggest that while in men hypersensitivities might be more important in the aetiology of sexual dysfunctions, in women it might rather be hyposensitivities. Women with ASD might need more intense sexual stimulation to become and stay sexually aroused during having sex and to reach an orgasm, explaining the lower sexual excitation scores. However, the ASD women in the present study also reported more frequently about sexual pain problems, suggesting that not only hyposensitivities but also hypersensitivities could be of relevance and it could be possible that normotypical sexual intercourse is perceived as painful by some ASD women. Both women with ASD and female controls scoring higher on sexual excitation reported better sexual functioning. Comparably, previous research found that in women from the general population higher SES scores were positively correlated with a more positive attitude towards sexuality, higher overall sexual functioning, higher sexual desire, higher sexual arousal, less problems with lubrication and higher orgasm quality [36,43,44]. Although women with ASD had significantly lower sexual inhibition scores than female HCs, no association was found between sexual inhibition and sexual functioning in the ASD women. ASD women have an up to three times increased risk to be sexually victimized than non-ASD women, which could explain the higher sexual inhibition scores. It could have been expected that those individuals with an especially pronounced propensity of sexual inhibition would also show more sexual dysfunctions, however, this was obviously not the case [45].

The findings of the present study are limited because diagnoses were assessed via self-report and one cannot be sure that all participants were diagnosed by a trained psychologist or psychiatrist. Due to data protection regulations we were not allowed to contact the diagnosing clinicians in order to verify the diagnoses of our study participants. We tried to reduce false positives by using the well-established cut-off of the German version of the AQ-SF, which proved in other studies to be sufficiently sensitive and specific to assess autistic symptomatology [27]. Nevertheless, future studies should choose a more standardized assessment approach concerning the verification of clinical diagnoses, for example by conducting a clinical interview. Furthermore, all participants were recruited through ASD self-help groups or ASD outpatient care centres, indicating that their contact with the medical system was due to their symptomatology. Although we assessed comorbid psychiatric disorders in general, we did not evaluate specific disorders, such as depressive or anxiety disorders, which are highly prevalent in autistic individuals and could affect sexual well-being and functioning. Using diagnostic interviews in future studies could help to also prevent this shortcoming. Furthermore, we did not assess intellectual functioning of our participants (e.g., by assessing IQ scores), however, as our study participants had on average 12 years of school education it can be assumed that all participants possessed at least average intellectual abilities. It is possible that especially individuals with a higher interest in sexuality-related issues and perhaps also with more sexual problems, were more likely to volunteer to participate in the present study leading to a sampling bias and an overestimation of sexual problems. However, it is likely that this should have also accounted for the individuals in the control group, thereby equalizing a possible overestimation of the actual rate of sexual dysfunctions in the ASD group at least to some degree. Our results are further limited by the fact that we did not evaluate whether or not the spouses of our ASD individuals were diagnosed with ASD as well. As stated above previous research has suggested higher sexual and relationship satisfaction when both companions are diagnosed with ASD. Thus, future studies addressing sexual functioning of ASD individuals should definitely consider this point. Finally, we did not evaluate hormonal profiles of our study participants, although differences in hormone serum concentrations could have a great impact on sexual functioning as well. Future studies should therefore assess the hormonal profiles of ASD individuals in order to find out if the increased prevalence of sexual dysfunctions found in ASD individuals is due to somatic or psychiatric reasons or both. At least though we did not find any differences in the self-reported frequency of endocrine disorders, genital abnormalities or hormonal substitution treatment.

The present study has shown that a considerable number of individuals with ASD report about a general relationship and sexual dissatisfaction and about sexual dysfunctions. Furthermore, the sexual problems are probably to a large part attributable to the disorder-specific symptoms, such as impaired social and interpersonal skills, difficulties in perspective taking and theory of mind and the peculiarities in sensitive perception. This points out that there is a great need for specialized treatment programs teaching individuals with ASD how they can, despite their disorder, have a fulfilling and satisfying sexual life. Unfortunately, such treatment programs are almost non-existent up to now, at least for adults with high-functioning ASD.

## Figures and Tables

**Table 1:** Sociodemographic and clinical characteristics of study participants.

	ASD (*n* = 96)	HCs (*n* = 96)
Male (*n*, %)	56 (58.3%)	57 (59.4%)
Age (years, SD)	39.2 (9.5)	37.9 (9.7)
School education (years, SD)	11.9 (1.5)	12.4 (1.3)
AQ sum score (M, SD)	26.7 (4.9)	6.4 (3.3)
Regular use of alcohol (*n*, %)	21 (21.9%)	50 (52.1%) **
Regular use of illegal drugs (*n*, %)	7 (7.3%)	12 (12.5%)
Any psychiatric disorders other than ASD	34 (35.4%) **	0
Endocrine disorders	5 (5.2%)	0
Genital abnormalities	0	0
Regular medication intake	57 (59.4%) **	16 (16.7%)
Regular intake of psychopharmacological drugs	30 (31.3%) **	2 (2.1%)
Hormone replacement therapy	4 (4.2%)	2 (2.1%)

ASD = Autism Spectrum Disorder; HCs = Healthy Controls. Regular intake is defined as at least three times a week. ** *p* ≤ 0.01.

**Table 2:** Average questionnaire sum and subscale scores compared between autism spectrum disorder (ASD) patients and healthy controls (HCs).

	ASD	HCs	*t/*χ^2^	*p*
Women				
SES	10.06 (SD = 4.14)	14.6 (SD = 2.67)	3.66	0.001
SIS1	11.94 (SD = 1.80)	9.53 (SD = 1.41)	−4.23	0.0001
SIS2	14.22 (SD = 1.66)	12.71 (SD = 1.94)	−2.37	0.03
FSFI Sum score (max. 95)	38.21 (SD = 22.65)	78.67 (SD = 9.48)	10.31	0.0001
FSFI Desire (max. 10)	3.3 (SD = 1.95)	6.27 (SD = 1.53)	7.52	0.0001
FSFI Arousal (max. 20)	8.5 (SD = 6.05)	16.87 (SD = 2.42)	8.03	0.0001
FSFI Lubrication (max. 20)	10.1 (SD = 7.83)	18.33 (SD = 1.68)	6.42	0.0001
FSFI Orgasm (max. 15)	6.45 (SD = 4.97)	12.80 (SD = 2.51)	7.14	0.0001
FSFI Satisfaction (max. 15)	6.95 (SD = 3.47)	11.93 (SD = 2.46)	7.34	0.0001
FSFI Pain (max. 15)	4.25 (SD = 6.49)	12.47 (SD = 4.19)	6.67	0.0001
Men				
SES	17.5 (SD = 3.20)	13.89 (SD = 2.53)	−4.56	0.0001
SIS1	10.04 (SD = 2.25)	9.56 (SD = 1.85)	−0.86	0.40
SIS2	12.67 (SD = 2.75)	11.96 (SD = 2.46)	−0.97	0.34
IIEF sum score (max. 75)	39.96 (SD = 14.35)	55.70 (SD = 19.11)	6.89	0.0001
IIEF erectile function (max. 30)	15.54 (SD = 7.56)	23.19 (SD = 9.50)	4.73	0.0001
IIEF orgasmic function (max. 10)	8.0 (SD = 3.19)	8.3 (SD = 3.12)	0.05	0.61
IIEF sexual desire (max. 10)	6.69 (SD = 1.98)	7.07 (SD = 1.57)	1.13	0.26
IIEF intercourse satisfaction (max. 15)	2.12 (SD = 4.11)	9.52 (SD = 4.58)	9.03	0.0001
IIEF overall satisfaction (max. 10)	4.65 (SD = 1.83)	7.63 (SD = 2.36)	7.49	0.0001
Below cut off for erectile function problems (<14)	12 (21.4%)	5 (8.8%)	3.54	0.06

FSFI = Female Sexual Function Index, IIEF = International Index of Erectile Functioning.

**Table 3:** Linear logistic regression addressing the relationship between clinical factors and sexual dysfunctions in individuals with Autism Spectrum Disorder (ASD).

	Coefficients				
	b	*SE*	*p*	Exp(B)	95% CI
**ASD Men**					
Regular alcohol intake	0.01	7.14	0.96	0.37	−14.53–15.26
Regular intake of illegal drugs	0.25	11.55	0.27	13.02	−11.07–37.10
Any other psychiatric disorder	0.04	6.43	0.86	1.15	−12.25–14.56
Regular intake of drugs	0.01	6.87	0.99	0.06	−14.28–14.40
Regular intake of psychopharmacological drugs	−0.51	8.19	0.08	−15.15	−32.24–1.94
**ASD Women**					
Regular alcohol intake	0.1	19.51	0.79	5.21	−37.74–48.16
Regular intake of illegal drugs	0.24	25.44	0.51	17.32	−38.68–73.32
Any other psychiatric disorder	0.03	14.58	0.93	1.40	−30.70–33.50
Endocrinological disorders	−0.50	22.86	0.21	−30.30	−80.61–20.02
Regular intake of drugs	0.37	20.34	0.30	22.36	−22.40–67.13
Regular intake of psychopharmacological drugs	−0.46	16.26	0.23	−20.65	−56.43–15.13
Hormone replacement therapy	−0.08	14.88	0.81	−3.76	−36.51–28.99

**Table 4:** Correlational analysis between the sexual inhibition/sexual excitation scales short form (SIS/SES) and female sexual functioning assessed with the female sexual function index (FSFI).

	FSFI Sum Score	FSFI Desire	FSFI Arousal	FSFI Lubrication	FSFI Orgasm	FSFI Satisfaction	FSFI Pain
ASD							
SES	0.40 **	0.48 **	0.56 **	0.43 **	0.51 **	−0.16	0.11
SIS1	−0.19	−0.19	−0.27	−0.26	−0.29	0.20	−0.14
SIS2	−0.26	−0.11	−0.20	−0.20	−0.09	0.06	−0.14
Healthy controls							
SES	0.58 **	0.50 **	0.30	0.13	0.45 **	0.64 **	0.63 **
SIS1	−0.27	−0.04	−0.13	−0.26	−0.15	−0.13	−0.26
SIS2	−0.12	−0.46 **	−0.43 **	−0.12	−0.03	−0.07	0.22

** *p* < 0.01.

**Table 5:** Correlational analysis between the SIS/SES and male sexual functioning assessed with the IIEF.

	IIEF Sum Score	IIEF Erectile Function	IIEF Orgasmic Function	IIEF Sexual Desire	IIEF Intercourse Satisfaction	IIEF Overall Satisfaction
ASD						
SES	0.19	0.21	0.17	0.10	0.18	0.01
SIS1	−0.37 **	−0.40 **	−0.31 *	−0.10	−0.37 **	−0.16
SIS2	−0.34 **	−0.36 **	−0.26 *	−0.37 **	−0.34 **	−0.04
Healthy controls						
SES	0.43 **	0.45 **	0.10	0.46 **	−0.01	0.30 *
SIS1	−0.01	−0.03	−0.23	0.06	0.14	0.07
SIS2	0.02	0.06	−0.21	−0.05	0.17	−0.03

* *p* < 0.05; ** *p* < 0.01.
